# Case Report: Expanding the *LRP5-*phenotypic spectrum of the novel c.4462A > G (p.Ser1488Gly) variant with isolated retinal involvement

**DOI:** 10.3389/fped.2026.1912905

**Published:** 2026-07-28

**Authors:** Mirjana Bjeloš, Ana Ćurić, Benedict Rak, Mladen Bušić, Katja Rončević, Adrian Elabjer

**Affiliations:** 1 University Eye Department, Reference Center of the Ministry of Health of the Republic of Croatia for Pediatric Ophthalmology and Strabismus, Reference Center of the Ministry of Health of the Republic of Croatia for Inherited Retinal Dystrophies, Reference Center of the Ministry of Health of the Republic of Croatia for Standardized Echography in Ophthalmology, University Hospital “Sveti Duh”, Zagreb, Croatia; 2 Faculty of Medicine, Josip Juraj Strossmayer University of Osijek, Osijek, Croatia; 3 Faculty of Dental Medicine and Health Osijek, Josip Juraj Strossmayer University of Osijek, Osijek, Croatia; 4 University Department of Nursing of the Catholic University of Croatia, Zagreb, Croatia; 5 School of Medicine of the Catholic University of Croatia, Zagreb, Croatia; 6 University of Zagreb School of Medicine, Zagreb, Croatia

**Keywords:** case report, familial exudative vitreoretinopathy, *LRP5*, variant of uncertain significance, Wnt signaling

## Abstract

**Background:**

*LRP5-*related retinal disease is classically associated with familial exudative vitreoretinopathy (FEVR), whereas biallelic inactivating variants cause osteoporosis-pseudoglioma syndrome. We report a severe congenital FEVR-like retinal dysplasia in a child carrying the novel heterozygous *LRP5* c.4462A > G (p.Ser1488Gly) variant of uncertain significance (VUS), adding carefully documented phenotypic layer to a ClinVar entry that has lacked disease-specific clinical context.

**Case presentation:**

A full-term female infant presented with nystagmus, absent visually directed behavior, and visual responsiveness limited to bright light. Examination under general anesthesia at 15 months revealed bilateral macular pseudocoloboma, mid-peripheral termination of retinal vascularization with avascular far peripheral retina, scattered pigment clumping, and microphthalmia. Optical coherence tomography showed marked outer retinal atrophy with an irregular V-shaped foveal contour, and electroretinography demonstrated non-detectable rod and cone responses. A targeted inherited retinal dystrophy panel identified the heterozygous *LRP5* c.4462A > G variant, absent from population databases and classified as a VUS. No pathogenic or likely pathogenic variants were found in other tested genes relevant to FEVR, Norrie disease spectrum, vitreoretinal dysplasia, microphthalmia-associated retinopathy, or early-onset retinal dystrophy. The variant was inherited from an asymptomatic father. No retinal intervention was indicated. Follow-up to 3 years showed no progression and no systemic features of *LRP5*-related bone disease.

**Conclusion:**

The phenotype is best interpreted as an isolated, atypical FEVR-like congenital retinal dysplasia within *LRP5-*associated FEVR spectrum. However, paternal inheritance from an unaffected carrier, atypical severity, and lack of functional validation preclude definite causal assignment. This report supports cautious VUS interpretation and adds clinically relevant phenotypic evidence for future reinterpretation of LRP5 p.Ser1488Gly.

## Introduction

The *LRP5* (low-density lipoprotein receptor-related protein 5) gene encodes a protein that is localized to the plasma membrane of various cell types, where it functions as a co-receptor in conjunction with frizzled-4, a receptor encoded by the *FZD4* gene. Together, these proteins mediate signal transduction through the canonical Wnt/*β*-catenin signaling pathway, involved in regulating cellular processes such as proliferation, adhesion, migration, and differentiation (1).

Heterozygous *LRP5* mutations are more often associated with autosomal dominant FEVR, typically in the absence of the systemic bone abnormalities (2, 3). In contrast, OPPG, is an autosomal recessive condition caused by biallelic *LRP5* inactivating mutations, resulting in congenital blindness, along with severe early-onset osteoporosis (4).

Outside the retina, *LRP5* is a key regulator of bone density. It modulates osteoblast activity through the canonical Wnt/*β*-catenin signaling pathway (5), and certain gain-of-function mutations in *LRP5* lead to high bone mass disorders (6). Conversely, loss-of-function variants result in reduced bone mass; homozygous null mutations cause the osteoporosis of OPPG (4, 5, 7), while some heterozygous missense variants have been linked to idiopathic osteoporosis (8).

We present a case of a young child carrying a novel heterozygous *LRP5* c.4462A > G (p.Ser1488Gly) VUS, who manifested atypical FEVR-like retinal dysplasia including macular pseudocoloboma, and no evidence of systemic *LRP5-*related abnormalities. This variant is absent from population databases and has not previously been reported in association with disease.

## Case presentation

### Patient information

The patient was a full-term female infant, the only child of healthy, non-consanguineous parents, born after an uneventful pregnancy and delivery, with non-contributory family history. In early infancy, nystagmus and absent visually directed behavior were observed, with visual responsiveness limited to bright light. No dysmorphic features were noted apart from deep-set orbits. Growth and overall health were normal. Follow-up to age three years demonstrated no clinical progression. No evidence of neurological deficit or global developmental delay, apart from the visual dysfunction, has been identified.

### Clinical findings

**Figure 1:**
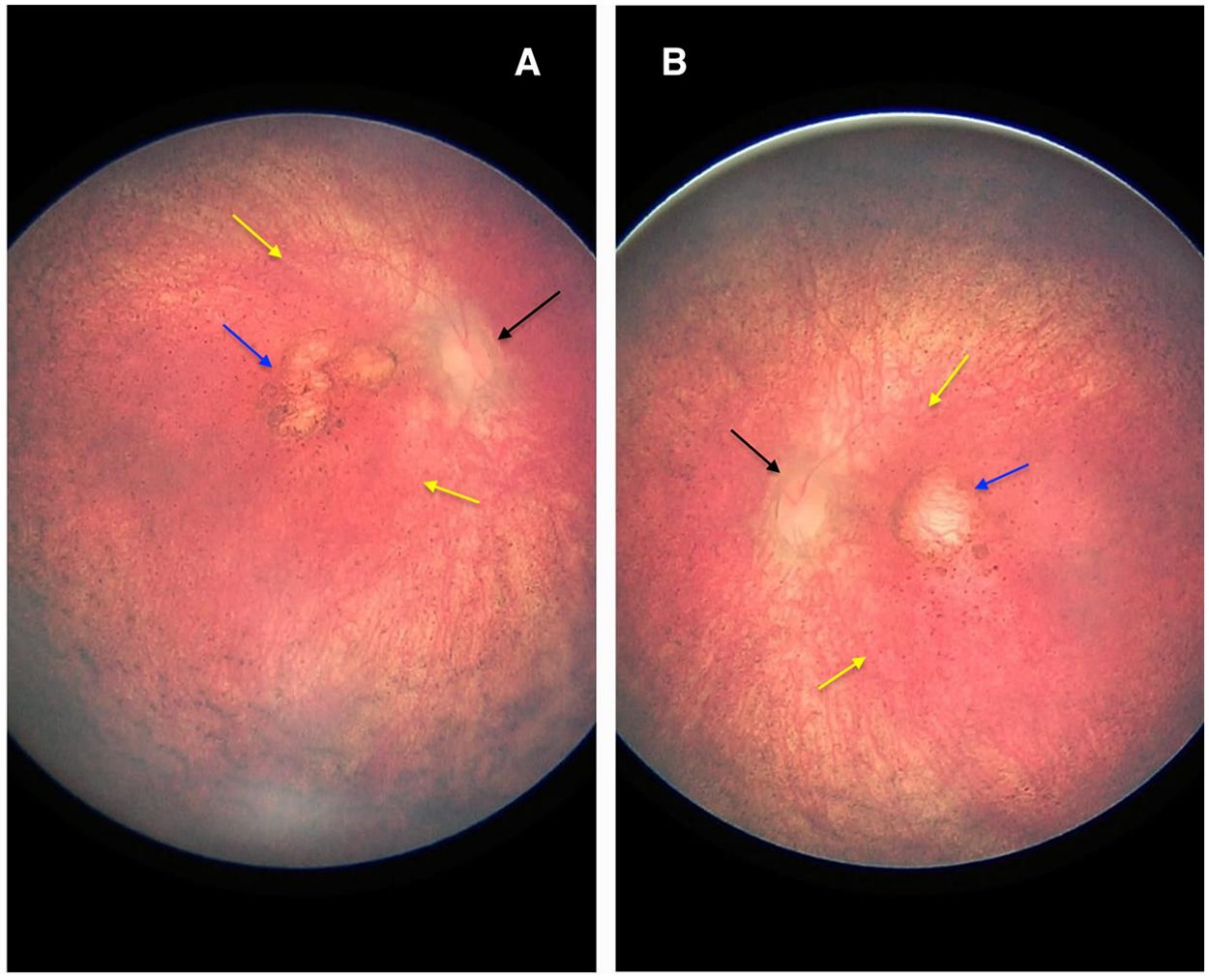
Retcam® ophthalmic imaging system of the right eye **(A)** and left eye **(B)** demonstrated sharply demarcated optic nerve (black arrows), excavated lesion in the central macula, consistent with a macular pseudocoloboma (blue arrows), and abruptly terminating retinal vascularization (yellow arrows).

A comprehensive ophthalmologic evaluation under general anesthesia was conducted at 15 months of age. RetCam® Envision (Natus Medical Inc., Middleton, Wisconsin, USA) imaging demonstrated a macular pseudocoloboma, with retinal vascularization abruptly terminating in the mid-periphery, leaving the far peripheral retina entirely avascular, with scattered pigment clumping. A-scan echography (AvisoTM ultrasound, Quantel medical by Lumibird medical, Cournon d'Auvergne, France) measured axial lengths of 17.66 mm in the right eye and 18.26 mm in the left eye, consistent with microphthalmia. There was no evidence of retinal detachment or neovascular proliferative membranes (Figure 1).

**Figure 2:**
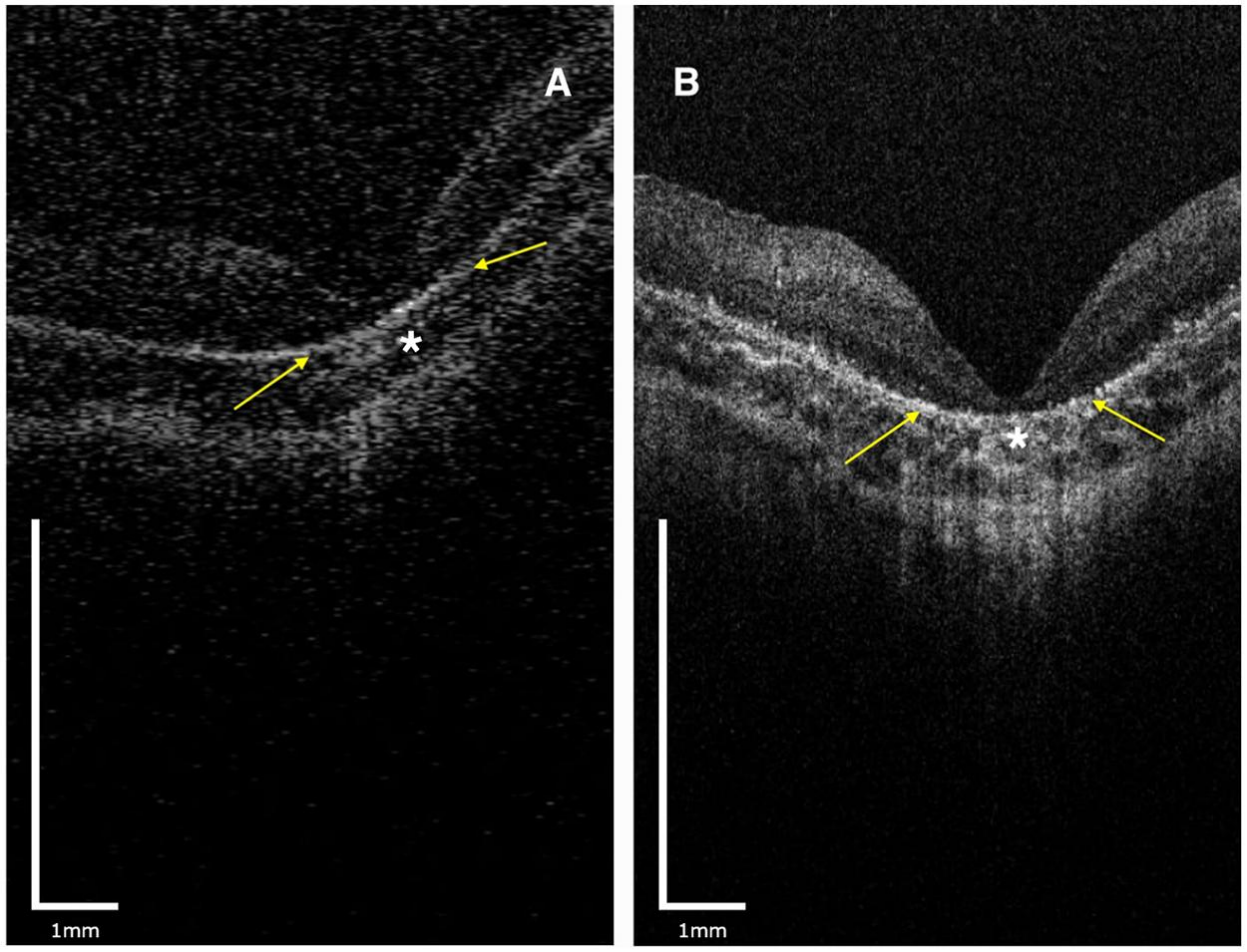
Spectral-domain optical coherence tomography of the right eye **(A)**, and left eye **(B)** revealed outer retinal atrophy (yellow arrows) with irregular V-shaped foveal contour and increased signal transmission in the choroid (white asterisks).

Spectral-domain optical coherence tomography (OCT) [Leica Envisu C-Class (C2300) spectral domain ophthalmic imaging system, Wetzlar, Germany] of the macula revealed marked thinning of the outer retinal layers in the foveal region, consistent with a coloboma-like atrophic defect (Figure 2).

An electroretinogram (ERG) (Roland Consult RETIport/scan 21, Roland Consult Stasche and Finger GmbH–German Engineering, Brandenburg an der Havel, Germany) was performed as a separate examination, without sedation or general anesthesia, and yielded non-detectable rod and cone responses.

### Timeline

Key clinical, imaging, and genetic events are summarized in Table 1.

**Table 1:** Timeline of key clinical, imaging, genetic, and follow-up events.

Time point	Event/assessment	Key findings or outcome
Prenatal/birth	Pregnancy, delivery, and family history	Full-term female infant; uneventful pregnancy and delivery; healthy non-consanguineous parents; non-contributory family history.
Early infancy	Initial visual concerns	Nystagmus and absent visually directed behavior were observed; visual responsiveness was limited to bright light.
15 months	Examination under general anesthesia and RetCam® imaging	Bilateral macular pseudocoloboma; retinal vascularization terminated in the mid-periphery with far peripheral avascular retina and scattered pigment clumping; no retinal detachment or neovascular proliferative membranes.
15 months	A-scan echography	Axial length measured 17.66 mm in the right eye and 18.26 mm in the left eye, consistent with microphthalmia.
15 months	Spectral-domain OCT	Marked outer retinal atrophy/thinning in the foveal region with an irregular V-shaped foveal contour and coloboma-like atrophic defect.
15 months	Electroretinography	Rod and cone responses were non-detectable.
Genetic testing	Targeted inherited retinal dystrophy gene panel	Single heterozygous LRP5 NM_002335.4:c.4462A > G, p.(Ser1488Gly) variant identified and classified as a VUS; no pathogenic or likely pathogenic variants detected in other relevant genes.
Parental testing	Segregation and paternal retinal assessment	Variant absent in the mother and present in the clinically asymptomatic father; ultra-widefield fundus examination and HRA+OCT® of the father were normal.
Post-diagnosis	Therapeutic management	No retinal intervention indicated; management consisted of observation, parental and genetic counseling, age-appropriate visual habilitation, and planned ophthalmologic surveillance.
Follow-up to 3 years	Clinical course and systemic assessment	No clinical progression; growth and overall health remained normal, without bone fragility or systemic features suggestive of OPPG. Motor and cognitive development were age-appropriate.

### Diagnostic assessment

A targeted inherited retinal dystrophy gene panel, the Blueprint Genetics Retinal Dystrophy Panel Plus Analysis (version 7, October 30, 2021), including both sequence and copy-number variation analysis of 314 nuclear genes and 37 mitochondrial genome genes associated with retinal disease, identified a single heterozygous variant in *LRP5* [NM_002335.4:c.4462A > G, p.(Ser1488Gly)] (9). No pathogenic or likely pathogenic (LP) variants were detected in in genes relevant to FEVR, Norrie disease spectrum, vitreoretinal dysplasia, microphthalmia-associated retinopathy, or early-onset retinal dystrophy, including *CTNNA1, ZNF408, ATOH7, RCBTB1, JAG1, KIF11, NDP, FZD4, TSPAN12*, *COL2A1, OTX2, RBP4, MFRP, RDH11* or *CRB1*. This *LRP5* variant was absent from the gnomAD population database, and in silico predictive tools yielded conflicting results (PolyPhen-2: benign, SIFT: tolerated, Mutation Taster: disease causing). The variant has not been previously reported in literature. ClinVar entry lists it as a VUS (10). The affected residue is highly conserved, providing moderate evidence toward pathogenicity, however, no functional validation or published disease association is currently available. Parental genotyping revealed that the variant was absent in the mother but present in the father, who remained clinically asymptomatic. Ultra-widefield fundus examination (Optos® California, Dunfermline, UK) and HRA+OCT ® (Heidelberg Engineering, Heidelberg, Germany) did not reveal any abnormalities in the father.

### Therapeutic intervention

No disease-modifying retinal intervention was performed because there was no retinal detachment, active exudation, or neovascular proliferation at the time of evaluation. Management consisted of observation, parental counseling, genetic counseling, age-appropriate visual habilitation, and planned ophthalmologic surveillance.

### Follow-up and outcomes

Follow-up to 3 years of age showed no clinical progression. Growth and overall health remained normal, and there was no clinical evidence of bone fragility or other systemic features suggestive of OPPG. The child demonstrated age-appropriate motor and cognitive abilities, as well as advanced verbal skills, including bilingual language acquisition.

## Discussion

The present case is best regarded as an isolated retinal phenotype within the spectrum of autosomal dominant *LRP5*-associated FEVR, complicated by incomplete penetrance, as it fulfills the key vascular feature of FEVR-spectrum disease: incomplete peripheral retinal vascularization with abrupt mid-peripheral vascular termination and bilateral avascular far peripheral retina (2, 11–14). No alternative retinal or systemic gene defect was identified within the tested panel, making *LRP5* the most biologically plausible candidate gene. However, because testing was limited to a targeted retinal dystrophy panel, an alternative or additional molecular diagnosis cannot be fully excluded, including deep intronic, regulatory, structural, or novel gene variants that would require broader genomic approaches such as whole genome or whole exome sequencing. The severity and configuration of the phenotype, particularly the coexistence of macular pseudocoloboma, microphthalmia, and extinguished ERG responses, are therefore atypical for conventional FEVR (14) and warrant cautious interpretation as an *LRP5*-associated FEVR-like congenital retinal dysplasia rather than a definitive molecular diagnosis.

To the best of our knowledge, this is the first report providing detailed phenotype-linked documentation of the currently minimally annotated ClinVar VUS *LRP5* c.4462A > G (p.Ser1488Gly). At present, the variant is listed in ClinVar under *not provided* (10), without an associated disease-specific phenotype or published affected individual. By documenting its occurrence in a child with severe congenital FEVR-like retinal dysplasia, while also emphasizing paternal inheritance from an unaffected carrier and the absence of functional validation, this report contributes cautiously annotated case-level evidence that may support future variant reinterpretation as additional cases, segregation data, or functional studies become available.

The retina is often described as a “Wnt-sensitive organ” because it requires precisely timed Wnt signaling for angiogenesis and neuronal patterning (1). During a narrow developmental window, Norrin, a glia derived ligand, must activate LRP5 on endothelial cells: if this signal is even moderately reduced, the vasculature fails to expand (11). After this window, however, later processes of vascular and neuronal maintenance may not rely as heavily on continued LRP5 signaling. This may explain why our patient's retinal phenotype is congenital and appeared relatively stable over the three-year follow-up period: vascular arrest and consequent photoreceptor compromise likely occurred early in development, as reflected by extinguished ERG responses. Importantly, the non-detectable rod and cone responses are not typical of classic FEVR with isolated peripheral avascularity, and this electrophysiological severity is one of the reasons why we interpret the phenotype as an atypical FEVR-like congenital retinal dysplasia rather than conventional FEVR. Animal models harboring loss-of-function mutations in *LRP5* exhibit incomplete retinal vascularization, characterized by a sparse superficial network and complete absence of the deep and peripheral plexuses (1). The metabolically demanding developing macula, deprived of its supporting deep capillary network, is prone to atrophic remodeling (1, 11, 15) and subsequent development of pseudocoloboma (16).

In *LRP5*-related FEVR, most pathogenic variants are missense changes that reduce, but do not eliminate Wnt signaling. This residual LRP5 activity may be insufficient to sustain normal retinal vessel growth yet remain adequate to support bone development. Humans also express *LRP6*, a paralog of *LRP5* that shares ∼73% sequence homology and exhibits overlapping functional roles in Wnt signaling (17). Notably, LRP5 (but not LRP6) is the co-receptor for Norrin-induced signaling in retinal vasculature (1). Consequently, an *LRP5* mutation affecting the eye cannot be functionally rescued by *LRP6*, as LRP6 is not known to fully compensate in Norrin-mediated signaling. In bone, Wnts (such as Wnt3a, Wnt10b) can signal through both LRP5 and LRP6: therefore, when LRP5 signaling is partially reduced, LRP6-mediated signaling may provide functional compensation (17). Double deficiency of *LRP5* and *LRP6* in the embryonic mesenchyme leads to a near-complete absence of osteoblasts and severe skeletal patterning defects (18). In our patient, one wild-type *LRP5* allele remains, and *LRP6* is presumably intact; this redundancy likely preserves sufficient Wnt signaling for osteoblast function and normal bone mass accrual (18).

The phenotypic discordance between the severely affected child and her clinically asymptomatic father, both heterozygous for the *LRP5* c.4462A > G (p.Ser1488Gly) variant, illustrates the well-documented phenomenon of incomplete or reduced penetrance and variable expressivity, whereby some carriers exhibit minimal or no retinal changes compared with the severe proband phenotype, in autosomal dominant *LRP5*-related FEVR (13, 19).

The LRP5 protein comprises 1615 amino acids and features a large extracellular domain containing four *β*-propeller/EGF-like repeat domains and multiple LDL receptor (LDLR)-type repeats, followed by a single transmembrane segment and a ∼200-residue intracellular cytoplasmic tail (5, 20). Within intracellular domain, five tandem “PPPSP” motifs (Pro-Pro-Pro-Ser-Pro), embedded in the larger consensus sequence PPPSPxS, are evolutionarily conserved and essential for downstream signal transduction (21). Mechanistically, upon Wnt ligand engagement with the Frizzled-LRP5/6 receptor complex, the PPPSPxS motifs become phosphorylated on serine/threonine (S/T) residues by casein kinase 1 (CK1) and glycogen synthase kinase 3β (GSK-3β) (22). This phosphorylation generates high-affinity docking sites for Axin, a scaffold protein that carries GSK-3β and other components of the β-catenin destruction complex (17). The binding of Axin to phosphorylated LRP5/6 results in its sequestration at the plasma membrane, effectively preventing it from targeting β-catenin for degradation (17). As a result, β-catenin accumulates in the cytoplasm and translocates to the nucleus, where it activates Wnt target gene expression (17). Functionally, each PPPSP motif acts as a molecular switch: when phosphorylated, it enables Wnt/β-catenin signaling. Experimental studies have demonstrated that mutating all five PPPSP motifs abolishes LRP6-mediated signaling, whereas even a single transplanted PPPSP motif is sufficient to confer Wnt signaling competence to an unrelated protein (3).

The variant p.Ser1488Gly is a missense change in the intracellular domain, lying within the conserved S/T-rich segment that precedes PPPSPxS motif A in LRP5 (23). Although specific phosphorylation sites within the upstream region of LRP5 have not been mapped, its strong positional and sequence conservation with LRP6, whose intracellular signaling architecture has been more extensively characterized, suggests a comparable regulatory framework (17).

**Figure 3:**
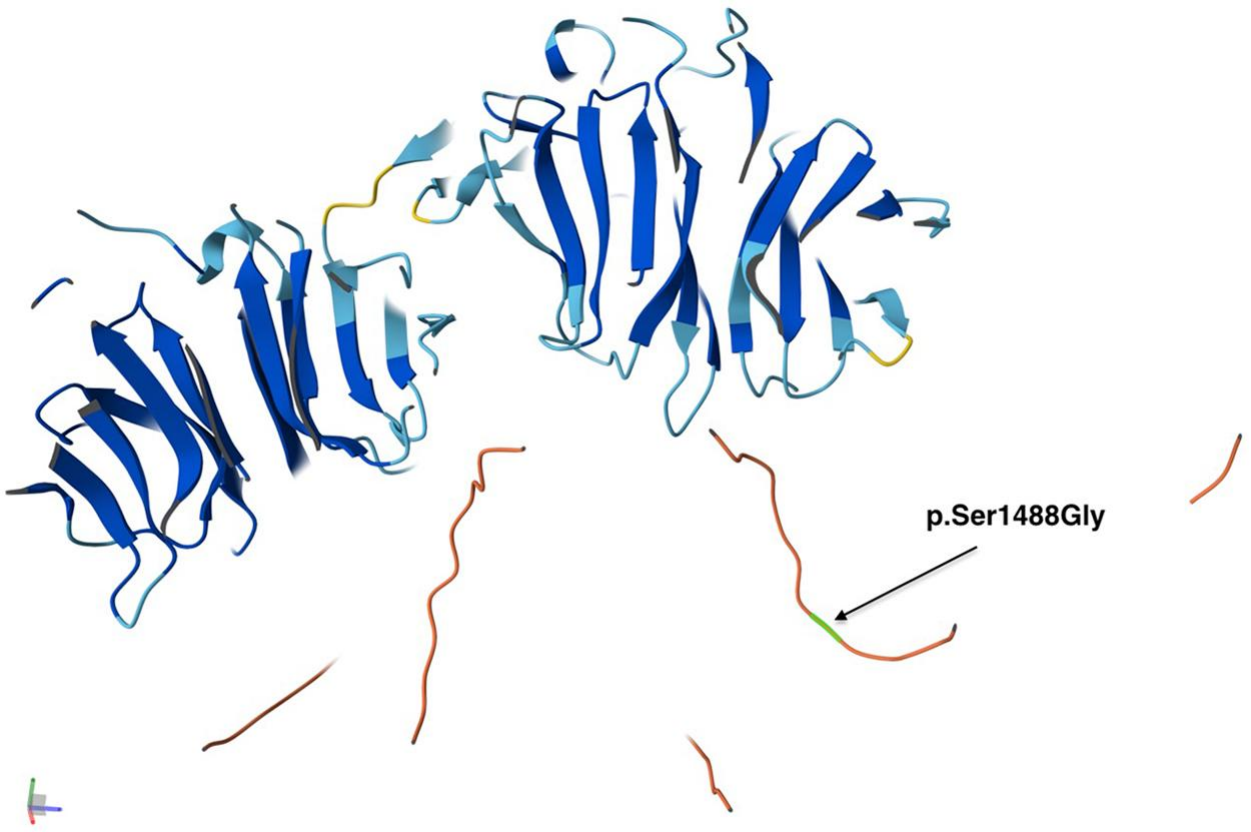
Alphafold – a predicted model of the LRP5 cytoplasmic domain.

Consistent with prior observations, AlphaFold (AF-O75197-F1) predicts uniformly low confidence and an absence of stable tertiary structure (Figure 3), compatible with a phosphorylation-rich interaction platform (24). Replacement of serine with glycine eliminates the hydroxyl group required for phosphorylation and introduces greater backbone flexibility, potentially disrupting conformational permissiveness that help orient the region for kinase recruitment and reducing the ability of the S/T-rich cluster to prime motif A phosphorylation. Early phosphorylation of the first motifs typically occurs rapidly and serves to nucleate further phosphorylation events (25). If the Ser1488Gly substitution reduces the efficiency of motif A phosphorylation, the overall number of phosphorylated PPPSPxS motifs may be diminished, leading to weaker Axin recruitment and a blunted Wnt response. Although the loss of a single upstream regulatory site would be expected to produce only a moderate reduction in signaling output, even partial loss-of-function can have disproportionate effects in biological contexts requiring maximal Wnt activity, such as retinal vascular development.

Pathogenic mutations causing OPPG frequently cluster in the extracellular *β*-propeller domains that mediate ligand binding, whereas missense variants associated with FEVR are more widely distributed, including within the cytoplasmic tail (3, 5). Notably, a nearby *LRP5* variant, p.A1525T, has been reported in individuals with early-onset osteoporosis, and several additional tail variants in the same study were predicted to be deleterious (26). Collectively, these findings reinforce the concept that single-residue alterations within the intrinsically disordered cytoplasmic domain may impair Wnt signaling. In this context, the homology between LRP5 and LRP6, together with the established priming role of the upstream S/T rich cluster, provides a mechanistic rationale for attributing hypomorphic properties to the p.Ser1488Gly variant, with possible selective impact on retinal Wnt signaling. However, because the *LRP5* c.4462A > G (p.Ser1488Gly) variant does not currently meet the evidence threshold for reclassification from VUS to LP (27) for retinal vascular disease within the Wnt/Norrin signaling pathway, and because segregation and functional data remain insufficient, its contribution to the patient's congenital FEVR-like vitreoretinopathy remains biologically plausible but unproven.

At present, the lack of clinically evident skeletal involvement can be interpreted in light of preserved canonical Wnt signaling capacity. Residual LRP5 function from the unaffected allele, together with intact LRP6-mediated signaling, may be sufficient to sustain osteoblast activity and early bone mass accrual (18). The retinal phenotype, however, points to a more stringent tissue-specific requirement for LRP5, as Norrin-driven signaling in retinal vasculature depends on LRP5 rather than on LRP6 as the relevant co-receptor (1). Thus, the predominantly ocular presentation may reflect the limited redundancy of the LRP5/Norrin axis in the retina, rather than a generalized failure of Wnt-dependent skeletal regulation. However, preventive measures regarding osteoporosis have been initiated, including ensuring adequate calcium and vitamin D intake and encouraging age-appropriate weight-bearing activity. One published family with a tail variant (A1537T) in LRP*5* had multiple members with osteoporosis in mid-life (28). However, given the patient's age of only 3 years, the absence of clinically evident osteoporosis should be interpreted cautiously, as reduced bone mass or impaired peak bone mass accrual may become apparent later in life.

*KIF11*- and *NDP*-related disorders were considered because both may mimic FEVR through abnormal retinal vascular development, peripheral avascularity, and congenital vitreoretinal dysgenesis. However, *KIF11*-associated disease was considered unlikely because the patient had no microcephaly, lymphedema, developmental delay, or intellectual disability, and *KIF11* sequence and copy-number analysis were negative (29). Similarly, the phenotype lacked retrolental pseudoglioma, persistent fetal vasculature (PFV) stalk, total retinal detachment, progressive hearing loss, seizures, or neurodevelopmental features typical of Norrie disease or severe *NDP*-related PFV (30). Female sex alone does not exclude *NDP*-related disease, but in the absence of an *NDP* molecular finding this diagnosis is substantially less likely (30).

*COL2A1*-related Stickler syndrome type 1 was included in the differential diagnosis because *COL2A1* variants are a major cause of inherited vitreoretinopathy and rhegmatogenous retinal detachment; however, the absence of the characteristic ocular and systemic features of Stickler syndrome, together with negative *COL2A1* testing, provided little support for this diagnostic category (31). Broader syndromic microphthalmia/coloboma disorders remain conceptually relevant because of the reduced axial length, but the overall phenotype did not fit this diagnostic group, given the absence of classical uveal coloboma, anterior segment or lens abnormalities, optic nerve malformation, dysmorphism, neurological impairment, or systemic anomalies (32).

One of the limitations of the study is that fluorescein angiography (FA) was not performed. However, given the severely avascular and atrophic retina and the absence of clinical signs of exudation or neovascular activity, FA was unlikely to provide additional diagnostic or therapeutic information in this context. Although the child was already under general anesthesia, FA would still have represented an additional invasive procedure requiring intravenous dye administration, without a clear expected impact on diagnosis or management.

From a broader developmental perspective, the clinical findings support a predominantly ocular phenotype rather than a syndromic neurodevelopmental presentation. In this case, no evidence of neurological deficit or global developmental delay, apart from the visual dysfunction, has been identified. The child demonstrated age-appropriate motor and cognitive abilities, as well as advanced verbal skills, including bilingual language acquisition. These findings suggest that the identified *LRP5* variant does not exert a deleterious effect on cerebral structures beyond the retina, in alignment with the current understanding of Wnt signaling biology, wherein LRP5 and its ligand Norrin are not regarded as major regulators of cortical neurodevelopment or cognitive function.

## Conclusion

In conclusion, this case highlights a severe congenital FEVR-like retinal dysplasia in a child carrying the novel heterozygous VUS *LRP5* c.4462A > G (p.Ser1488Gly). The phenotype, characterized by macular pseudocoloboma, microphthalmia, peripheral avascular retina, and extinguished electroretinographic responses, expands the clinical context in which this currently minimally annotated ClinVar variant has been observed (1). At the same time, paternal inheritance from an unaffected carrier, the atypical severity of the ocular findings, and the absence of functional validation define the limits of current interpretation. Rather than establishing a new disease-causing variant, this report adds a carefully documented phenotypic layer to a ClinVar entry that has, until now, lacked disease-specific clinical context. Such cautiously annotated observations are particularly relevant in pediatric rare disease genetics, where individual institutions often cannot accumulate sufficient phenotypic, segregation, and functional evidence alone. Transparent case-level reporting of this kind may help pool evidence, support future reinterpretation of rare variants, and reduce the risk of premature overinterpretation of VUS findings in diagnostically complex children.

## Patient perspective

The patient was too young to provide a first-person perspective. The parents/legal guardians provided written informed consent for publication of the deidentified clinical findings and clinical images and were counseled regarding the uncertain significance of the genetic result, the need for ongoing follow-up, and the possibility that broader genomic testing may be informative.

## Data Availability

The datasets presented in this article are not readily available because of ethical and privacy restrictions. Requests to access the datasets should be directed to the corresponding author.
